# Analysis of Allogenicity of Mesenchymal Stem Cells in Engraftment and Wound Healing in Mice

**DOI:** 10.1371/journal.pone.0007119

**Published:** 2009-09-22

**Authors:** Liwen Chen, Edward E. Tredget, Chenxiong Liu, Yaojiong Wu

**Affiliations:** 1 College of Animal Science, South China Agricultural University, Wushan, Guangzhou, China; 2 Life Science Division, Tsinghua University Graduate School at Shenzhen, The University Town, Shenzhen, China; 3 Department of Surgery, Department of Biochemistry, University of Alberta, Edmonton, Alberta, Canada; Centre de Recherche Public de la Santé (CRP-Santé), Luxembourg

## Abstract

Studies have shown that allogeneic (allo-) bone marrow derived mesenchymal stem cells (BM-MSCs) may enhance tissue repair/regeneration. However, recent studies suggest that immune rejection may occur to allo-MSCs leading to reduced engraftment. In this study, we compared allo-BM-MSCs with syngeneic BM-MSCs or allo-fibroblasts in engraftment and effect in wound healing. Equal numbers of GFP-expressing allo-BM-MSCs, syngeneic BM-MSCs or allo-fibroblasts were implanted into excisional wounds in GFP-negative mice. Quantification of GFP-expressing cells in wounds at 7, 14 and 28 days indicated similar amounts of allogeneic or syngeneic BM-MSCs but significantly reduced amounts of allo-fibroblasts. With healing progression, decreasing amounts of allogeneic and syngeneic BM-MSCs were found in the wound; however, the reduction was more evident (2 fold) in allo-fibroblasts. Similar effects in enhancing wound closure were found in allogeneic and syngeneic BM-MSCs but not in allo-fibroblasts. Histological analysis showed that allo-fibroblasts were largely confined to the injection sites while allo-BM-MSCs had migrated into the entire wound. Quantification of inflammatory cells in wounds showed that allo-fibroblast- but not allo-BM-MSC-treated wounds had significantly increased CD45^+^ leukocytes, CD3^+^ lymphocytes and CD8^+^ T cells. Our study suggests that allogeneic BM-MSCs exhibit ignorable immunogenicity and are equally efficient as syngeneic BM-MSCs in engraftment and in enhancing wound healing.

## Introduction

Bone marrow derived mesenchymal stem cells (BM-MSCs), which are also referred to as stromal progenitor cells, are self-renewing and expandable stem cells. Numerous studies have suggested that they are of potential therapeutic value. Transplantation of *ex vivo* expanded allogeneic (allo-) BM-MSCs improves repair to the infarcted heart [1] and brain [2] and enhance wound healing [3] in animals. Allogeneic BM-MSCs derived from healthy donors have been used to treat diseases in humans [4], [5]. In addition, MSCs have interesting immunologic properties *in vitro*. They have been demonstrated by multiple investigators to suppress stimulated T cells in co-culture experiments [6]–[12], although the mechanisms are not fully understood. These *in vitro* observations suggest that MSCs may evade alloimmune surveillance, induce specific immunologic tolerance, and suppress graft-versus-host-disease (GVHD) [4], [13], [14]. However, controversial results were shown in recent *in vivo* studies [15], [16]. Subcutaneously implanted MSCs engineered to release erythropoietin to allogeneic mice were found to cause shorter lasting increase in hematocrit than to syngeneic mice, and allogeneic MSC implants had an increased proportion of host-derived lymphoid CD8^+^, natural killer T (NKT), and NK infiltrating cells compared with syngeneic controls [15], suggesting that immune reaction to allo-MSCs may caused reduced cell engraftment. When allo-MSCs were added to a bone marrow transplant, they yielded no clinical benefit on the incidence or severity of GVHD. However, the absence of clinical effect was shown not due to MSC rejection because they still could be detected in grafted animals [17]. Therefore, whether allogeneic MSCs have reduced engraftment and therapeutic effect than autologous MSCs needs to be elucidated.

In this study, we compared allo-BM-MSCs with syngeneic BM-MSCs or allo-fibroblasts in engraftment and effect on the healing of excisional wounds in mice. Our data demonstrated similar engraftment patterns and enhancements in wound healing between allogeneic and syngeneic BM-MSCs, though decreasing amounts of engrafted cells were found in both types of MSCs with progression of the wound healing process. However, reduction in number of allo-fibroblasts in the wound was much more dramatic (2 fold) which was associated with no improvement in wound closure. Analysis of inflammatory cells in the wound indicated that wounds treated with allo-fibroblasts but not allo-BM-MSCs had significantly increased amounts of CD45^+^ leukocytes, T lymphocytes and CD8^+^ T cells. Our data suggest that allo-BM-MSCs do not cause immune inflammation and are as effective as syngeneic cells in enhancing wound healing.

## Methods

All animal procedures were approved under the guidelines of the Health Sciences Animal Policy and Welfare Committee of the University of Alberta.

### Isolation, purification and characterization of MSCs

The bone marrow was collected from the femurs and tibia of 5–7 week-old male C57-GFP transgenic mice (C57BL/6 TgN[ACT6EGFP, Jackson Laboratory) and nucleated cells were isolated with a Ficoll-paque density gradient. The nucleated cells were plated in plastic tissue culture dishes and incubated in minimal essential medium (α-MEM; Invitrogen) supplemented with 17% fetal bovine serum (FBS). When reaching 80% confluent, the adherent cells were harvested and subjected to immunodepletion using antibody-coated magnetic micro beads (Miltenyi Biotec) against CD34, CD14, Gr1, CD3 and CD19. Characterization of the cells for their immunophenotypic markers by fluorescent-activated cell sorting (FACS) showed that they were negative for cell lineage markers CD45, CD14, CD34, CD19, CD3, Flk-1 and positive for typical MSC surface proteins Sca-1, CD105, CD29 and CD44 [14]. After culturing in induction media [18], [19], the cells differentiated into adipocytes, osteoblasts and chodrocytes. Passage 3–5 cells were used for the experiments.

### Isolation of dermal fibroblasts

The skin of 5–7 week old GFP mice (C57BL/6 TgN[ACT6EGFP] was incubated with Dispase I (Sigma) in keratinocyte-SFM (Invitrogen) at 10 mg/ml for 13 hours at 4°C to remove the epidermis. Fibroblasts were obtained from the dermis after digestion with 0.75% collagenase and cultured in DMEM supplemented with 10% FBS. Passage 3–5 cells were used for the experiments.

### Flow cytometry

Excised wounds together with a small amount of the surrounding skin were dispersed enzymatically into single cell suspensions as previously described [20]. In brief, the tissue was incubated with dispase I at 1 mg/ml overnight at 4°C, minced and incubated in a digestion buffer containing hyaluronidase (1 mg/ml), collagenase D (1 mg/ml) and DNase (150 units/ml) (Sigma) in a 37°C shaking water bath for 2 hours. The dispase and the hyaluronidase digests were pooled and filtered through a 70 um Nylon cell strainer. Cells were washed, pelleted, resuspended in PBS containing 3% FBS. Murine blood was collected by cardiac puncture. Peripheral blood mononucleated cells (PBMCs) were isolated by density gradient centrifugation with Ficoll-Hypaque. 100 µL cell aliquots each containing at 1×10^5^ cells were first blocked with Mouse BD Fc Block and then incubated with phycoerythrin (PE)-conjugated monoclonal antibodies specific for CD45, Gr-1, CD14, CD3, CD8a (BD Pharmingen), or control isotype IgG on ice for 30 minutes. After washing with PBS, the samples were analyzed by flow cytometry (Becton Dickinson) using Cell Quest software.

### Wound healing model

**Figure 1:**
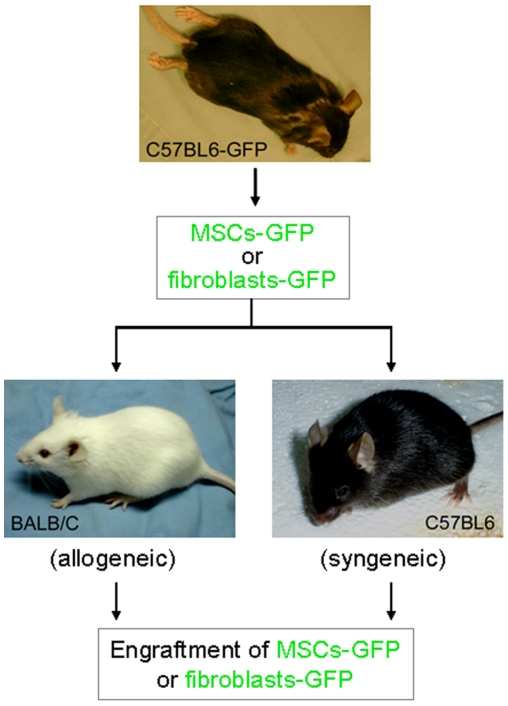
Experimental scheme.

GFP^+^ BM-MSCs or GFP^+^dermal fibroblasts derived from C57BL/6-GFP mice were transplanted to excisional wounds in Balb/C (allogeneic) or C57BL/6 mice (syngeneic) (Figure 1). Balb/C or C57BL/6 mice (8 week-old, female, body weight 19–23 grams, Jackson Laboratory) were randomly divided into groups (n = 21) and the excisional wound splinting model was generated as described previously [21]. In brief, after hair removal from the dorsal surface and anesthesia, two 5-mm full-thickness excisional skin wounds were created on each side of the midline. Each wound received one million cells (GFP^+^ BM-MSCs or GFP^+^dermal fibroblasts derived from C57BL/6-GFP mice): 0.7×10^6^ in 60 µl PBS injected intradermally around the wound at 4 injection sites and 0.3×10^6^ in 20 µl Growth Factor Reduced (GFR) Matrigel (BD) applied onto the wound bed. A donut-shaped silicone splint was placed so that the wound was centered within the splint. An immediate-bonding adhesive (Krazy Glue®) was used to fix the splint to the skin followed by interrupted sutures to stabilize its position (Figure 1A) and Tegaderm (3 M) was placed over the wounds which was further covered by bandage (3 M bandaging type). The animals were housed individually. We tested the adhesive on the skin in mice prior to this experiment and did not observe any skin irritation or allergic reaction.

### Wound analysis

Digital photographs of wounds were taken at days 0, 3, 7, 10 and 14 days. Time to wound closure was defined as the time at which the wound bed was completely reepithelialized and filled with new tissue. Wound area was measured by tracing the wound margin and calculated using an image analysis program (NIH Image). The investigators measuring samples were blinded to group and treatment. The percentage of wound closure was calculated as: (area of original wound − area of actual wound)/area of original wound ×100. The inside edge of the splint exactly matched the edge of the wound, so that the splinted hole was used to represent the original wound size. Mice were sacrificed at 7 and 14 days when skin samples including the wound and 4 mm of the surrounding skin were harvested using a 10 mm punch biopsy. One wound which was bisected into two pieces and one of them was used for histology. The other wound was digested for FACS analysis.

### Immunostaining and confocal microscopy

Tissue specimens were fixed in 3% freshly prepared paraformaldehyde (PFA) for 24 h and embedded in OCT. Six-micron-thick tissue sections were pre-incubated with sodium borohydride (1 mg/ml in PBS) to reduce auto-fluorescence and then incubated with an monoclonal antibody against CD3 (R&D) which was followed by detection with a Cy3-conjugated secondary antibody. Nuclei were stained with Hoechst. Sections were examined with a Zeiss LSM 510 confocal microscope.

### Statistical analysis

All values are expressed as mean±SD. Student's paired *t* test was performed for comparison of data of paired samples and ANOVA was used for multiple group comparisons followed by Friedman's post test. A probability (*P*) value <0.05 was considered significant.

## Results

### Effect of allogeneic or syngeneic BM-MSCs in wound healing

**Figure 2:**
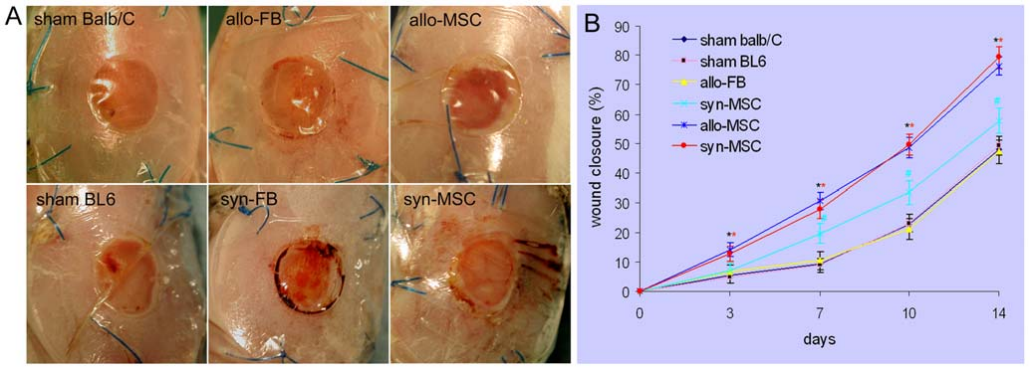
Effects of BM-MSCs on wound closure. (A) Excisional wounds in allogeneic (allo-) Balb/C or syngeneic (syn-) C57BL/6 mice received implantation of C57BL/6 derived GFP^+^ BM-MSCs (MSC), dermal fibroblasts (FB) or control vehicle medium (sham). Representative photographs of wounds at day 7 (with transparent Tegaderm dressing) are shown. Within the splint, the red area represents the unhealed area and the surrounding white area indicates the newly grown tissue from the wound edge. (B) Measurement of wounds (at day 3 and 7, n = 12 to 14; at day 10 and 14, n = 6 or 7). ANOVA, vs sham *P<0.01, # *P*<0.05.

We implanted equal numbers of BM-MSCs derived from C57BL/6-GFP mice into excisional wounds in Balb/C (allogeneic) or C57BL/6 (syngeneic) mice. Allogeneic or syngeneic BM-MSC-treated wounds exhibited accelerated wound closure compared to allogeneic dermal fibroblast- or vehicle medium-treated wounds (*P*<0.01, Figure 2A&B). The enhancement appeared early at 3 days and became more evident 7 days after cell implantation. No significant differences in wound closure were found between allogeneic and syngeneic BM-MSC-treated wounds. Syngeneic dermal fibroblasts, but not allogeneic fibroblasts, showed a modest effect in enhancing wound closure (*P*<0.05, Figure 2A&B).

### Engraftments of allogeneic or syngeneic BM-MSCs

**Figure 3:**
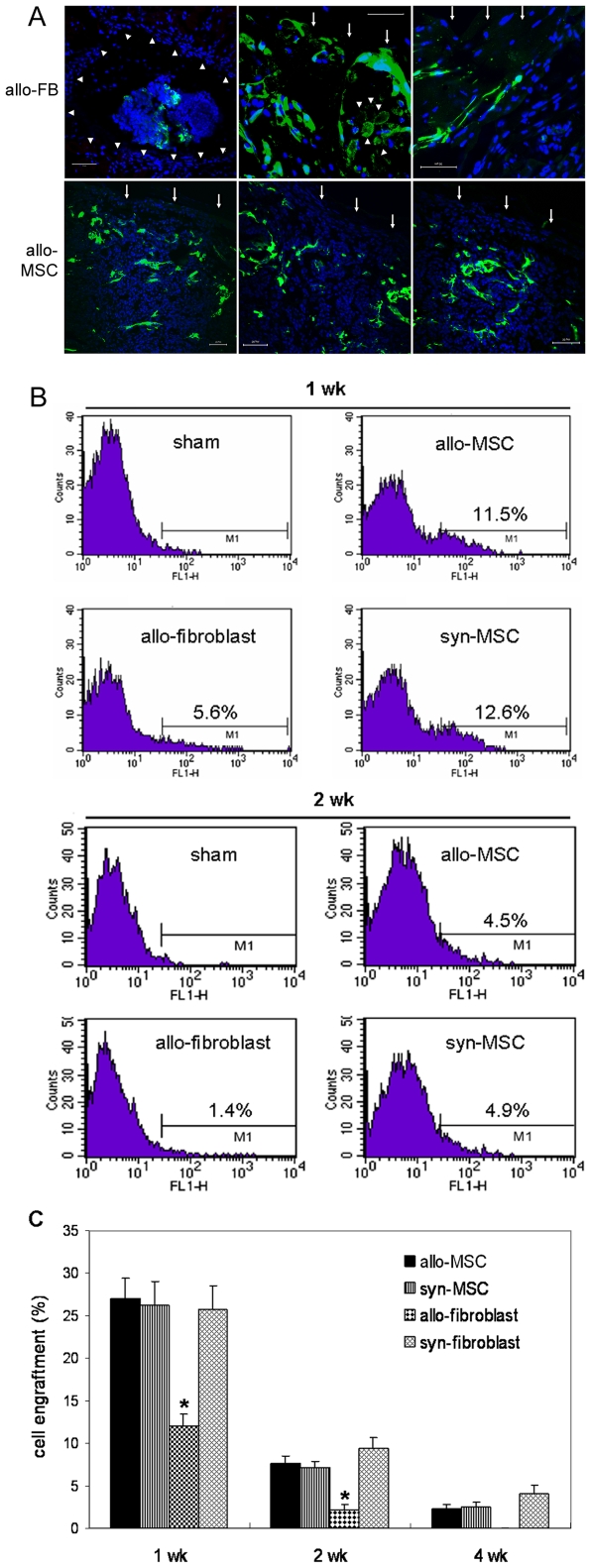
Engraftment of BM-MSCs into the wounded skin. (A) Allo-fibroblasts or allo-MSCs in wounds. Representative fluorescence microscopic images of day 7 wound sections showing that the injected allogeneic GFP^+^fibroblasts (allo-FB) were confined to the injection site and surrounded by a layer of inflammatory and fibroblast-like cells (arrow heads, left panel). Weak GFP signals were detected in some of allo-fibroblasts. After immunostaining for GFP, topically applied allo-fibroblasts (green) were shown to be poorly incorporated into the tissue (middle and right panels of upper row) and in many of them nuclei were not shown (arrow heads, middle panel of upper row), indicating cell death, while similarly applied allo-MSCs (green) were closely integrated into the wound (lower row, representative images from three mice). Wound beds are indicated by arrows. Nuclei were stained blue with Hoechst. scale bar, 50 µm. (B) Wounds treated with allogeneic or syngeneic BM-MSCs or vehicle medium (sham) in Balb/C or C57BL/6 mice at 1 or 2 weeks were enzymatically dissociated as discribed in “Materials and Methods” and single-cell suspensions were analyzed by flow cytometry to detect percentages of GFP-positive cells. One representative result is shown. Cells from sham wounds were used for negative controls and gate setting. (C) Cell engraftment. Taking the initially implanted one million cells per wound as 100%, proportions of engrafted BM-MSCs or fibroblasts at different times after transplantation are shown. *P<0.001 (allo-fibroblast vs MSC, n = 6 or 7).

To examine engraftments of BM-MSCs into the wound, we performed immunofluorescence analysis of tissue sections for GFP-expressing cells. At 7 days, abundant GFP-positive allogeneic or syngeneic BM-MSCs were found through out the wound which were closely associated with host cells in the tissue (Figure 3A). In contrast, allo-fibroblasts, but not syngeneic-fibroblasts were largely restricted to the injection sites where they were surrounded by a layer of inflammatory cells and host fibroblast-like cells. The topically applied fibroblasts on the wound bed largely failed to incorporate into the tissue and many of them lost their nuclei indicating cell death (Figure 3A). At 14 days, allogeneic or syngeneic GFP-MSCs were localized to the wound bed and skin appendages while only few GFP-fibroblasts were observed. At day 28, GFP-MSCs were rarely detected and no intact GFP-fibroblasts were found in the wound. In analysis of six animals per group at each time point, syngeneic BM-MSCs showed similar engraftment patterns to allogeneic BM-MSCs. To quantify GFP-MSCs or GFP-fibroblasts in wounds at different times, we excised the entire wound along with a small amount of the surrounding skin and dispersed it enzymatically into a single cell suspension. Counting of cells in the suspension with a cytometer resulted in the number of cells per wound (Table 1). Fractions of GFP-positive cells in the single-cell suspension were determined by FACS analysis (Figure 3B). Taking the initially implanted one million cells per wound as 100%, after calculation, proportions of engrafted BM-MSCs or fibroblasts at different times after transplantation were obtained (Figure 3C). Consistent with the findings in immunohistological analysis, similar amounts of allo-MSCs and syngeneic MSCs, but significantly fewer allo-fibroblasts were indicated in wounds at 1, 2 and 4 weeks (Figure 3C, n = 6 or 7, *P*<0.001). Different from allo-fibroblasts, similar amounts of syn-fibroblasts to syn-MSCs at 1 and 2 weeks, and greater amount of syn-fibroblasts than syn-MSCs at 4 weeks (Figure 3C, n = 6, *P*<0.05) were found in the wound.

**Table 1:** Number of cells per wound (×10^6^).

	sham (Balb/C)	sham (BL/6)	allo-FB	syn-FB	allo-MSC	syn-MSC
1 wk	1.9±0.22	1.8±0.18	2.2±0.29*	2.3±0.26^#^	2.7±0.27*	2.5±0.25^#^
2 wk	1.5±0.15	1.4±0.19	1.7±0.27	2.1±0.22^#^	2.1±0.23*	1.9±0.23^#^
4 wk	1.4±0.19	1.3±0.23	1.7±0.22*	1.5±0.24	1.4±0.20	1.2±0.21

The number of cells per wound was derived from counting of cells in a single cell suspension derived from digestion of an entire wound. Values represent means±SD, n = 6 or 7. In Balb/C recipient mice, * *P*<0.05 (vs sham Balb/C); in C57BL/6 recipient mice, ^#^ *P*<0.05 (vs sham C57BL/6).

### Inflammatory cells in wounds treated with allogeneic or syngeneic BM-MSCs

**Figure 4:**
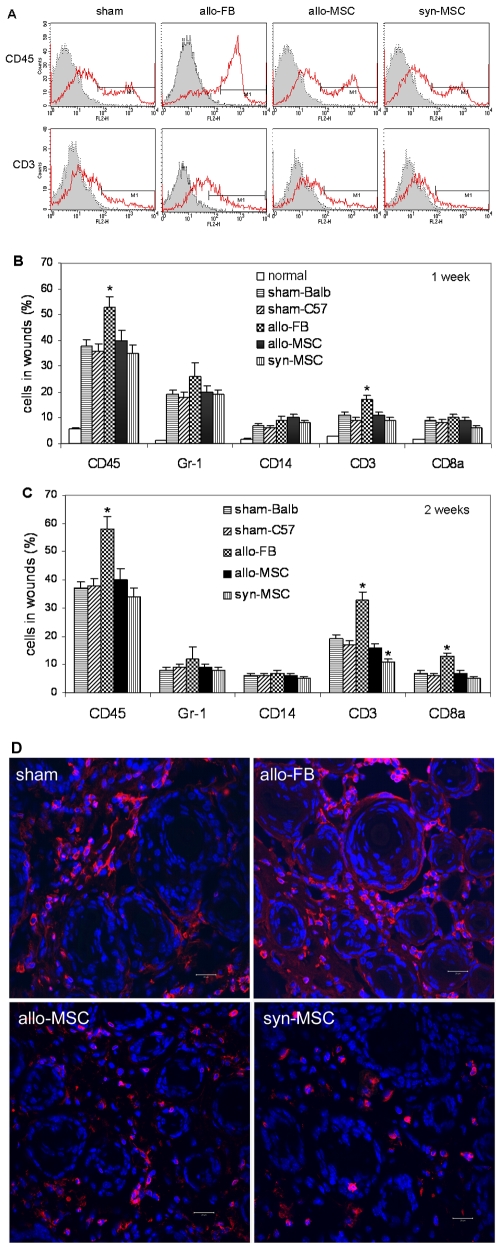
Leukocytes in wounds. Single-cell suspensions of the normal skin or wounds treated with allo-fibroblasts, allogeneic (allo-) or syngeneic (syn-) BM-MSCs or vehicle medium (sham) in Balb/C or C57BL/6 mice at 7 or 14 days were analyzed by flow cytometry after immunostaining. (A) Representative results of analysis of day 14 wound single cell suspensions for CD45^+^ leukocytes or CD3^+^ T cells (read peaks). Grey peaks represent negative controls. (B&C) Shown are means±SD of percentages of leukocytes or leukocyte subsets in the normal skin or wounds at 7 (B) or 14 (C) days (n = 6 or 7, *P<0.01). (D) Representative confocal microscopic images showing CD3^+^ T cells (red) in wounds at 14 days. Nuclei were stained blue with Hoechst. scale bar, 20 µm.

To examine whether allo-MSCs caused immune reaction, we quantified inflammatory cells in the single-cell suspension by FACS analysis. Samples were immuno-stained with monoclonal antibodies against CD45 (for leukocytes), Gr-1 (for granulocytes), CD14 (for monocytes), CD3 (for T lymphocytes) or CD8 (for CD8 T cells). Significantly increased leukocytes and T cells were found in wounds treated with allo-fibroblasts but not allo -BM-MSCs at 1 and 2 week compared to wounds treated with vehicle medium (Figure 4A–C). Moreover, CD8 T cells were also significantly increased in wounds received allo-fibroblasts at 2 weeks (Figure 4C). No significant differences in proportions of leukocytes, granulocytes, monocytes, T cells and CD8 cells were found between allo-MSC- and vehicle medium-treated wounds at both 1 and 2 weeks (Figure 4B&C, n = 6 or 7, *P*<0.01). However, significantly reduced CD3 T cells were found in syngeneic MSC-treated wounds (Figure 4C). Consistent with the findings in FACS analysis, immunofluorescence analysis showed that wounds treated with allo-fibroblasts had increased presence of CD3 T cells while wounds treated with syngeneic MSCs had reduced abundance of T cells compared to wounds treated vehicle medium (Figure 4D). To examine whether implantation of allogeneic MSCs or fibroblasts caused systemic changes in inflammatory cells, we quantified fractions of the above leukocyte subsets in the blood by FACS analysis, and found no significant differences compared to sham groups.

## Discussion

Following acute injuries such as myocardial infarction and cerebral strokes, on time delivery of BM-MSCs to the damaging tissues is crucial to achieve optimum therapeutic effects. However, autologous MSCs are technically unsuitable for such conditions due to delay in *ex vivo* cell expansion. Allogeneic BM-MSCs have been shown to improve structural and functional recoveries to the infarcted myocardium and brain in animals [1], [2], [4], [5]. Genetically modified allogeneic BM-MSCs to overexpress anti-apoptosis gene Akt could further augment the therapeutic effect of the cells to the infarcted heart [22]. These studies have suggested that allogeneic BM-MSCs are of potential value in cell therapies or cell-based gene therapies. However, recent studies suggest that immune rejection may occur to allo-MSCs leading to reduced graft survival [15], [16]. To examine whether autologous MSCs have a better engraftment and therapeutic effect, we implanted equal numbers of allogeneic or syngeneic BM-MSCs into excisional wounds in mice, and allo-fibroblasts and syn-fibroblasts were used as a controls. Our data indicated that allo-MSCs had similar engraftment and therapeutic effect in wound closure compared to syngeneic BM-MSCs. The numbers of allo-MSCs and syngeneic MSCs in the wound declined similarly with progression of the wound healing process. However, the reduction in the number of allo-fibroblasts in the wound was more evident than syn-fibroblasts. This result is consistent with our findings in immunohistochemical analysis of the wound where allo-fibroblasts, but not syngeneic-fibroblasts or allo-MSCs, were found to largely be confined to the injection sites by a layer of inflammatory cells and host fibroblast-like cells, and cause increased infiltration of CD3 T cells in the wound, displaying signs of immune reaction. In consistence with our observation, the amount of engrafted allogeneic MSCs in the infarcted myocardium was found to decrease dramatically at two weeks post transplantation [23]. Our data suggest that the reduction in the number of engrafted MSCs in healing wounds may largely be caused by changes in the microenvironment with progression of the wound healing process rather than immune reactions to MSCs. Consistent with our findings, in a recent study allogeneic BM-MSCs were found to be as efficient as syngeneic BM-MSCs in promoting wound healing [24]. It is likely that with progression of the wound healing process, cytokines and ECM molecules favorable to MSC survival and engraftment have decreased. It has been known that engraftment of BM-MSCs to normal non- hematopoietic tissues is extremely low.

In this study, we found significantly increased leukocytes, T cells and CD8 lymphocytes in wounds received allo-fibroblasts but not allo-MSCs. Injected allo-fibroblasts were confined to the injection site where they were surrounded by host inflammatory cells and fibroblast-like cells. In contrast, allo-MSCs had migrated into the entire wound. These results suggest that allogeneic fibroblasts but not allogeneic BM-MSCs cause immune inflammatory reactions which lead to reduced engraftment. In agreement with our results, increased inflammation and fibrosis were found in wound received allogeneic fibroblasts in previous studies [25], [26], but allo-MSCs could be detected in the infarcted myocardium two months post transplantation which were accompanied by decreased leukocyte infiltration in the tissue [1]. Moreover, allogeneic osteogenic cells derived from BM-MSCs were found to be able to survive *in vivo* despite expression MHC class II in the cells after differentiation [27]. Taken together, these data indicate that allogeneic MSCs have lower immunogenicity than dermal fibroblasts. The mechanisms underlying the differential immunogenicity of allogeneic MSCs and allogeneic dermal fibroblasts have not been fully understood. Previous studies suggest that the low immunogenicity of MSCs might attribute to their specific cell surface features, including low surface densities of MHC class I molecules and undetectable expression of MHC class II as well as costimulatory molecules such as CD80, CD86 and CD40 [6], [28]. However, dermal fibroblasts have also been known to have a similar expression pattern these cell surface molecules [25], [29]. Therefore, the surface antigenic properties appear unlikely to be an important mechanism for the differential immunogenicity of these two types of stromal cells. Instead, immunosuppressive activities of MSCs may play a more important role. Several studies have shown that MSCs suppress the proliferation and activities of a broad range of immune cells, including T cells, antigen-presenting cells, natural killer (NK) cells and B cells, probably through release of cytokines including indoleamine 2,3-dioxygenase, transforming growth factor beta-1, prostaglandin E, and nitric oxide [6], [28], [30]. Moreover, we found that MSCs and dermal fibroblasts differentially expressed numerous cytokines in our previous study. MSCs released higher levels of cytokines involved in cell growth and tissue repair/regeneration such as insulin-like growth factor-1, vascular endothelial growth factor-α, erythropoietin and stromal cell-derived factor-1, while dermal fibroblasts secreted greater amounts of pro-inflammatory cytokines such as interleukin-6 [31]. These data suggest that MSCs may posses special activities that suppress excessive inflammation and maintain homeostasis of the immune system through physical and/or chemical interactions with immune/inflammatory cells, thereby mediating host immune tolerance to allogeneic cells, particularly to themselves [30]. The molecular network for these cellular activities remains to be elucidated.

In contrast to most studies, a previous study showed that allo-MSC grafts had increased CD8 and NKT cells compared to syngeneic MSC grafts [15], suggesting that immunogenicity of allogeneic MSCs causes host immune rejection. Many factors may contribute to this controversial finding, such as the purity and identity of “MSCs” used in the study. Bone marrow cells adherent to plastic tissue culture dishes are highly heterogeneous particularly in mice, and often contain hematopoietic cells [32]. The cells used in the study were CD90^−^ and constitutively expressed MHC class II and CD80 [15], which were different from typical BM-MSCs, which have been defined as CD90^+^, and MHC class II- and CD80-negative cells [5], [14]. It is unclear whether these cells are different in allogenicity. In the present study, using purified BM-MSCs and a straight forward approach, we provide evidence to show that the immunogenicity of allogeneic BM-MSCs is very modest.

Previous studies suggest that the therapeutic effect of BM-MSCs in acute myocardial infarction occurs early within days following implantation which may largely be attributed to paracrine factors released by the cells [33], [34]. In this study, we found that enhancement in wound closure was evident as early as three days following administration of BM-MSCs. It suggests that BM-MSCs may mainly affect activities in early stages of the wound healing process such as cell recruitment and angiogenesis, and therefore early delivery of MSCs is crucial to achieve optimum effect in repair/regeneration.
