# Expression of Concern: Analysis of Allogenicity of Mesenchymal Stem Cells in Engraftment and Wound Healing in Mice

**DOI:** 10.1371/journal.pone.0300027

**Published:** 2024-02-29

**Authors:** The PLOS ONE Editors

After this article [1] was published, concerns were raised about results reported in Figs 3 and 4.

Specifically:

- In Fig 3A, there appears to be partial overlap between the allo-MSC middle panel and the allo-MSC right panel. These are described in the figure legend as showing representative results from different animals.
- In Fig 4A, the grey traces appear highly similar in the sham, allo-MSC, and Syn-MSC panels for the CD45 experiment, and in the allo-MSC and Syn-MSC panels for the CD3 experiment.

The corresponding author noted that the data underlying this article’s results are no longer available.

In response to the Fig 3A concern, the corresponding author stated that in their assessment the indicated panels appear similar but not the same. PLOS stands by our assessment and remains concerned that the two panels in question appear to report overlapping fields of the same data.

Regarding the Fig 4A concerns, the corresponding author stated that it is possible for control IgG peaks to be similar because control IgG is not immunized against any antigens and the allo- and syn-MSC treated wounds had no differences in healing. Another author noted that the same control aliquot was stained with the same IgG control for each individual surface marker, and stated:

“*The isotype IgGs for anti-CD45 antibody and for anti-CD3 antibody were different*, *the former was Lewis IgG2b, λ, and the later was Sprague-Dawley (outbred) IgG2b,* *κ. So the grey peaks for CD45 and CD3 look different in general. The grey peaks for* *Sham wound, Allo-MSC wound and Syn-MSC wound look similar than the one for* *Allo-FB in CD45 and CD3, likely because the Allo-FB wound had more severe* *inflammation than the other three, which potentially added a different background.* *The peaks in Allo-MSC and Syn-MSC look more similar because the two wounds showed no difference in healing*.”

PLOS obtained input on the Fig 4A issue from two Editorial Board members. Both agreed with the concerns about the similar control results. One Academic Editor advised that the high degree of similarity in the indicated control curves between treatment conditions indicates that the same cell suspension sample was used for the different control stainings, instead of using an aliquot of the corresponding treatment control sample. (Of note, there were also panels within each experiment for which control results differed from those listed above.) The Academic Editors advised that additional information about the control experiments should have been provided in the article, and that reuse of a control across experimental groups is not best practice for this type of experiment. However, an Academic Editor also commented that the Fig 4A concerns do not raise major concerns about the figure’s overall results because the wound cells are expected to be comparable with similar levels of autophorescence and nonspecific binding.

The above concerns cannot be fully resolved in the absence of the original data, but based on the editorial assessment they are unlikely to have major implications for the article’s overall conclusions. Therefore, the *PLOS ONE* Editors issue this Expression of Concern and advise readers to interpret the results in Figs 3A and 4A with caution.
